# The complete mitochondrial genome sequence of the medicinal fungus *Inonotus obliquus* (*Hymenochaetaceae*, Basidiomycota)

**DOI:** 10.1080/23802359.2019.1675548

**Published:** 2019-10-11

**Authors:** Retno Agnestisia, Akiko Ono, Luna Nakamura, Rei Chino, Kaito Nodera, Haruna Aiso-Sanada, Ikumi Nezu, Futoshi Ishiguri, Tomohiro Suzuki, Shinso Yokota

**Affiliations:** a United Graduate School of Agricultural Science, Tokyo University of Agriculture and Technology, Fuchu, Japan;; b School of Agriculture, Utsunomiya University, Utsunomiya, Japan;; c Center for Bioscience Research and Education, Utsunomiya University, Utsunomiya, Japan;; d Kyushu Regional Breeding Office, Forest Tree Breeding Center, Koshi, Japan;; e Department of Wood Properties and Processing, Forestry and Forest Products Research Institute, Tsukuba, Japan (Research Fellow of Japan Society for the Promotion of Science)

**Keywords:** *Inonotus obliquus*, medicinal fungus, mitochondrial genome, higher basidiomycetes

## Abstract

*Inonotus obliquus* is a medicinal fungus in the family *Hymenochaetaceae* and commonly known as chaga. The sclerotium of this fungus has been used as a traditional medicine for long time. In this study, we present the mitochondrial genome sequence of *I. obliquus*. The mitochondrial DNA (mtDNA) is 119,110 base pairs in length and contained genes for 58 Open reading frames, 2 ribosomal RNAs, and 30 transfer RNAs. Consequently performed phylogenetic analysis indicates that this fungus is closely related to *Sanghuangporus sanghuang* which belongs to the same family *Hymenochaetaceae.* We first reported about the complete mitochondrial genome of fungi belonging to the genus *Inonotus.*

*Inonotus obliquus* is a parasitic fungus that usually grows on birch and other trees. It is a higher basidiomycete belonging to the family *Hymenochaetaceae*. The sclerotium of this fungus, known as Chaga, has been used for centuries in Russia to treat cancer, cardiovascular disease and diabetes (Huang 2002) and has attracted attention due to its high medicinal value. It has a wide range of immunological properties such as antitumor (Taji et al. 2008), antioxidant (Cui et al. 2005), immunomodulatory (Staniszewska et al. 2017) and anti-asthma (Yan et al. 2011). To date, the mitochondrial DNA sequence of just one species, *Sanghuangporus sanghuang,* belonging to the family *Hymenochaetaceae* (to which *I. obliquus* also belongs) has been determined (Han et al. 2018). However, there is still very little knowledge about this family, hence further mitochondrial DNA (mtDNA) sequencing and phylogenetic analysis are needed. To analyse molecular evolution and phylogenetics in the family *Hymenochaetaceae,* we first determined the complete mtDNA of this fungus and then analysed its phylogenetic relationship with other basidiomycetes.

*I. obliquus* strain IO-B2 was collected in Nakagawa-gun, Hokkaido, Japan (44°33′14.7″N, 142°35′05.6″E; the collected specimen is stored in NITE Biological Resource Centre (NBRC), Japan, Accession number: NBRC113408). The genomic DNA of this fungus was extracted using the cetyltrimethylammonium bromide (CTAB) method (Doyle 1991; Tanaka et al. 2019). The genomic library was prepared using the TruSeq DNA PCR-free LT Sample Prep Kit and then sequenced it 2 × 301-base paired-end mode on the MiSeq (Illumina). The obtained paired-end reads were trimmed using Trimmomatic (Bolger et al. 2014) and khmer (Bankevich et al. 2012) to utilise only high quality reads. The resultant reads (3,572,325 pairs) were further assembled using SPAdes ver.3.11.1 (Bankevich et al. 2012). The mtDNA obtained was represented by a circular DNA molecule of 119,110 bp in length with a GC content of 25.0% (GenBank Accession No. LC497415). Its annotation was conducted using the MFannot tool (http://megasun.bch.umontreal.ca/cgi-bin/mfannot/mfannotInterface.pl) followed by manual curation. Ribosomal RNA genes were manually predicted by aligning to mtDNA of *Sanghuangporus sanghuang* (NC_039931) using Mauve alignment software (Darling et al. 2004). The mtDNA of *I. obliquus* contains 90 genes including 58 putative protein-coding genes, 2 ribosomal RNAs (*rnl* and *rns*), and 30 tRNAs (covered all 20 amino acids). The 58 protein-coding genes encode 14 conserved mitochondrial proteins of 3 cytochrome oxidases (cox1–3), apocytochrome b (cob), 7 NAD dehydrogenases (nad1–6 and nad4L), and 3 ATP synthases (atp6, atp8 and atp9).

Phylogenetic analysis using the amino acid sequences of 9 conserved proteins (cox1-3, cob, nad1-3 and nad5-6) was conducted using Geneious version 9.1 (Kearse et al. 2012), as described by Suzuki et al. (2019) and Tanaka et al. (2017). After alignment of these concatenated amino acid sequences using the MAFFT alignment software (Katoh et al. 2002), the phylogenetic analysis was conducted by the neighbor-joining method (Saitou and Nei 1987). The phylogenetic tree showed that *I. obliquus* is closely related to *S. sanghuang* belonging to the family *Hymenochaetaceae* (Figure 1).

**Figure 1.**
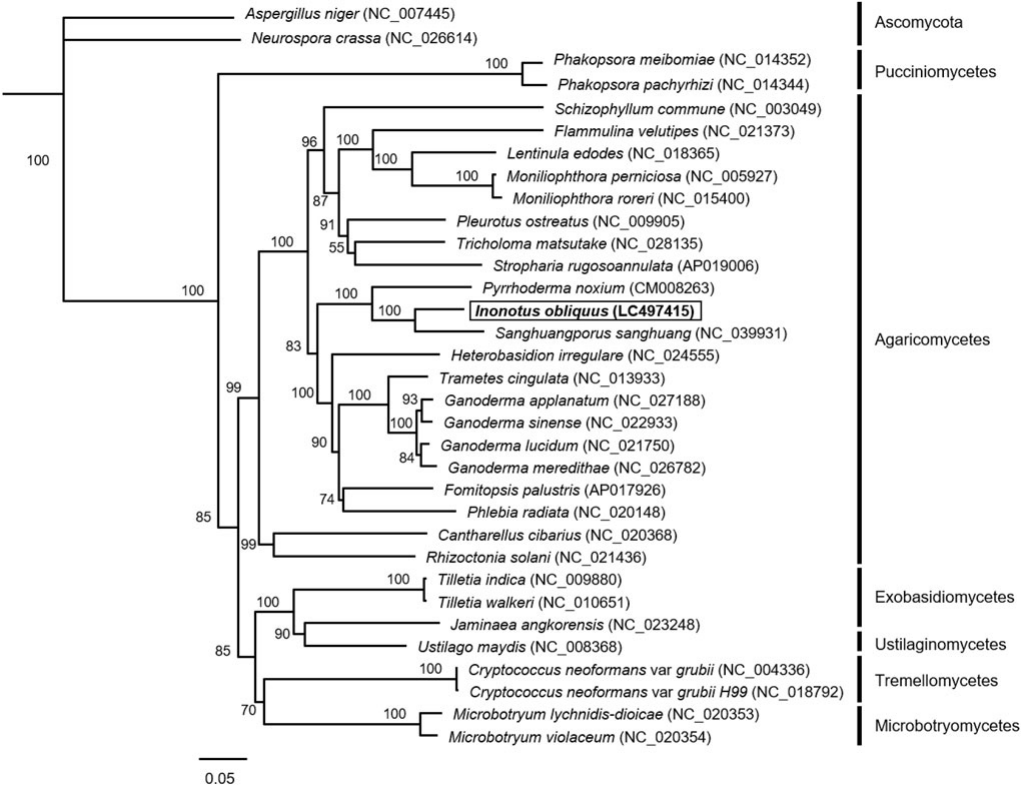
Phylogenetic relationships among 31 basidiomycetes and 2 ascomycetes. The phylogenetic analysis was conducted using concatenated amino acid sequences of 9 mitochondrial proteins (cox1-3, cob, nad1-3 and nad 5-6) by neighbor-joining method. Bootstrap values are shown at the nodes. Scale in substitutions per site.
